# Coronectomy as an alternative technique to complete extraction of mandibular third molars with risk of nerve injury

**DOI:** 10.4317/medoral.27459

**Published:** 2025-10-17

**Authors:** Laura Rico-Barroso, María-Encarnación Barroso-Medel, José-Luis Gutiérrez-Pérez, María-Ángeles Serrera-Figallo, Daniel Torres-Lagares

**Affiliations:** 1 University of Seville, Faculty of Dentistry. C/Avicena, s/n, 41009 Seville, Spain; 2 Dr Balmis University General Hospital, Av. Pintor Baeza 12, 03010, Alicante, Spain

## Abstract

**Background:**

Extraction of mandibular third molars (MTM) is one of the most frequent surgical procedures in Oral Surgery. This intervention is not without risks, as one of the most common is an injury of the inferior alveolar nerve (IAN). The main objective of this systematic review is to compare the coronectomy technique with conventional extraction of MTM with a high risk of injury to the IAN, thus determining whether coronectomy is an effective surgical alternative in preventing nerve injuries.

**Material and Methods:**

A bibliographical search was conducted in October 2024 in the Pub-Med and Cochrane-Database databases, and 211 studies were obtained. Finally, 16 studies were included in the systematic review, with a total sample of 2551 patients. 2280 coronectomy procedures and 841 extractions were performed.

**Results:**

6.22% of the coronectomies performed were unsuccessful. The incidence of IAN injury was 6.53% in the extraction group, while in the coronectomy group, it was only 0.56%. Permanent IAN injury was 1.18% and 0.13%, respectively. The most common complication in both groups was postoperative pain, with similar results, 15.69% in the extraction group vs. 14.21% in the coronectomy group. Lingual nerve injury was infrequent and transient in both groups, affecting 0.12% of extractions and 0.26% of coronectomies. The complication with the highest incidence in the coronectomy group was root migration, mainly during the first 6 months.

**Conclusions:**

Coronectomy is a safe and effective alternative technique for the management of MTM with a high risk of IAN injury.

## Introduction

Because 35.9% and 58.7% of the general population have at least one impacted third molar, impacted third molars, especially mandibular ones, are a relatively common cause of pain and inflammation in the oral region (1 , 2).

These impacted molars also have a well-documented associated pathology: Cysts, tumors, caries, and pericoronitis are among the most common pathologies, which leads to the need for extraction treatment in most of these cases (3).

Surgical extraction of mandibular third molars is a standard procedure; however, it is not exempt from postoperative complications, such as pain, trismus, swelling, and general oral discomfort, which usually subside within a few days. Furthermore, although less frequently, the anatomical proximity of the mandibular third molar (MTM) to the inferior alveolar nerve (IAN) canal is considered a risk factor for significant complications. Injury to the lingual nerve (LN) (incidence of damage between 0.1% and 22%) or the IAN (incidence of temporary sensory deficit from 0.41% to 8.1% or permanent from 0.0145% to 3.6%) is one of the most common causes of litigation in dentistry. These nerve disorders can cause neuralgia, paresthesia, and other alterations that may be temporary or permanent (2 - 4). In addition, seven radiological diagnostic signs are suggested as predictors of probable nerve damage: 4 signs at the tooth root (darkening, deviation, narrowing, and bifid apex) and 3 in the mandibular canal (CM) (deviation, narrowing, and interruption of the white line of the canal) (5 , 6). It is recommended to resort to TC / CBCT when these predictive clues appear in OPG, thus minimizing the incidence of nerve damage.

Among the surgical alternatives to MTM extraction when there is a risk of injury to the IAN is coronectomy. This technique, applied to impacted MTM, was first described by Ecuyer and Debien in 1984 and consists of the partial removal of the MTM, extracting its crown and leaving the apical 5-6mm of the roots in situ inside the mandibular alveolar bone (7 , 8). It was proposed to avoid injury to the IAN when extracting wisdom teeth whose roots were closely related to the CM. This surgical procedure is indicated for vertical, mesioangular, or strangulated MTMs with the involvement of the IAN, in which the crown section can be performed without damaging adjacent anatomical structures and whose complete extraction implies an increased risk of nerve damage. On the contrary, it is contraindicated in cases of caries or infection affecting the roots, in roots that have been mobilized during coronectomy maneuvers, in horizontal impactions on the CM, when it is unknown whether all the enamel of the tooth can be removed and if the second molars are intended to be visualized by orthodontic treatment (9 , 10).

Many studies have shown that coronectomy is an effective and safe alternative technique for preventing damage to the IAN when radiographic studies reveal anatomical proximity between the CM and the roots of the MTM. However, although rare, it is not without complications, with pain, infection, and root migration being some of the most common postoperative manifestations.

When planning therapeutic action for impacted wisdom teeth, the risk/benefit ratio must be assessed. Therefore, whether to perform the extraction, coronectomy, or even a wait-and-see approach will depend on multiple factors.

The primary objective of this study was to conduct a systematic review comparing coronectomy with complete MTM extraction in patients with high-risk signs of inferior alveolar nerve (IAN) injury.

The secondary objectives were:

1) To compare the incidence of IAN and LN injury in the coronectomy technique versus complete lower wisdom tooth extraction.

2) To evaluate the incidence of surgical complications such as postoperative pain, infection, dry socket, need for reoperation, and root migration in patients undergoing both coronectomy and complete MTM extraction.

3) To determine the number of failed coronectomy procedures.

## Material and Methods

Identification of study articles

Following the PRISMA guidelines, a bibliographic search for articles was conducted in October 2024 in the PubMed and Cochrane Databases to prepare this systematic review. The objective was to investigate the benefits of coronectomy over complete MTM extraction. The keywords used in the searches were a combination of: "("coronectomy" OR "partial odontectomy" OR "intentional root retention") AND ("mandibular third molar" OR "inferior third molar" OR "lower third molar")."

The research question was posed according to the PICO (population, intervention, comparison, outcomes) format: Does coronectomy reduce the incidence of nerve injury and postoperative complications compared with conventional total extraction in MTM surgery with a high risk of IAN injury? P (population): Patients with MTM at high risk of IAN injury. I (intervention): MTM surgery. C (comparison): Coronectomy vs conventional total extraction. O (outcomes): IAN and LN injury and postoperative complications.

Regarding the inclusion criteria, the following filters were applied: Articles published between 2005 and 2024, with full text in Spanish or English, and with cases referring to humans presenting with MTM at risk of nerve injury. Randomized controlled trials (RCTs), controlled clinical trials (CCTs), prospective and retrospective cohort studies (with or without a control group), and case-control studies were included. Included studies had to have a minimum sample size of 10 coronectomy cases and a follow-up period of at least 6 months. Case reports, in vitro studies, author comments, and systematic and literature reviews were excluded.

**Figure 1:**
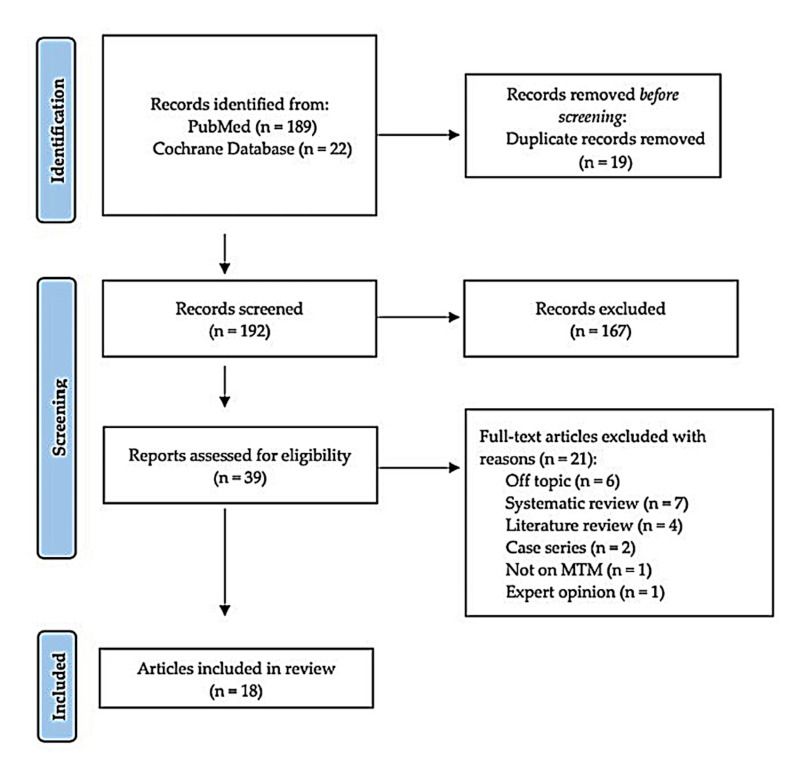
Flow diagram of literature search and selection criteria adapted from PRISMA.

The initial search yielded 211 studies. After applying filters, inclusion and exclusion criteria, and removing duplicates, the results were reduced to 39 studies. Finally, after reading the articles' titles and abstracts, those that were not relevant to the objectives were discarded, leaving a final selection of 16 articles for the study. Figure 1 illustrates a flowchart summarizing the entire process.

1

Study design

Finally, 16 studies were included in the systematic review, and all of them evaluated the following parameters: Number of patients, number of extractions and/or coronectomy procedures, and follow-up period. The most frequently reported complications in the included articles were also determined: Loss of sensation of the IAN and LN; the onset of pain, infection, or alveolitis; migration of the MTM roots after coronectomy and cases requiring reoperation, as well as failed coronectomies. The use or absence of pharmacological treatment was also indicated. In all studies, a p&lt;0.05 was considered statistically significant.

Risk of bias within studies

The risk-of-bias assessments for the individual studies are shown in Table 1. The Joanna Briggs Institute Critical Appraisal Tools for Prevalence Studies assessed the risk of bias. It was categorized as High when the study reached up to 49% scored "Yes," Moderate when the study reached 50 to 69% score of "Yes," and Low when the study reached more than 70% score of "Yes." In our systematic review, the vast majority of the studies reached up to 100% "Yes," meaning they have a very Low risk of bias. Only one of our studies was scored with Medium Risk.

Table 1

Supplementary Materials: Prisma Checklist (http://www.medicina.oral.com/carpeta/suppl1_27459)

## Results

To answer the proposed objectives, 16 studies (11 - 26) were analyzed in this systematic review (Table 2). 10 were prospective cohort studies, 3 were randomized controlled trials, 2 were case-control studies, and 1 was a retrospective cohort study. The articles included a total sample of 2551 patients. 2280 MTM coronectomy procedures and 841 complete MTM extractions were performed. All studies included at least a pre-operative panoramic radiography, except Mukherjee et al. (11), who only performed a periapical intraoral radiography.

Table 2

Patients were followed for a minimum of 6 months and 144 months.

**Figure 2:**
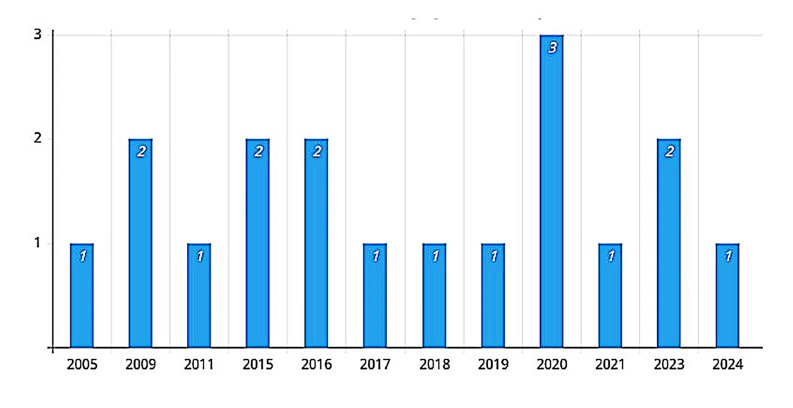
Number of articles by year of publication.

**Figure 3:**
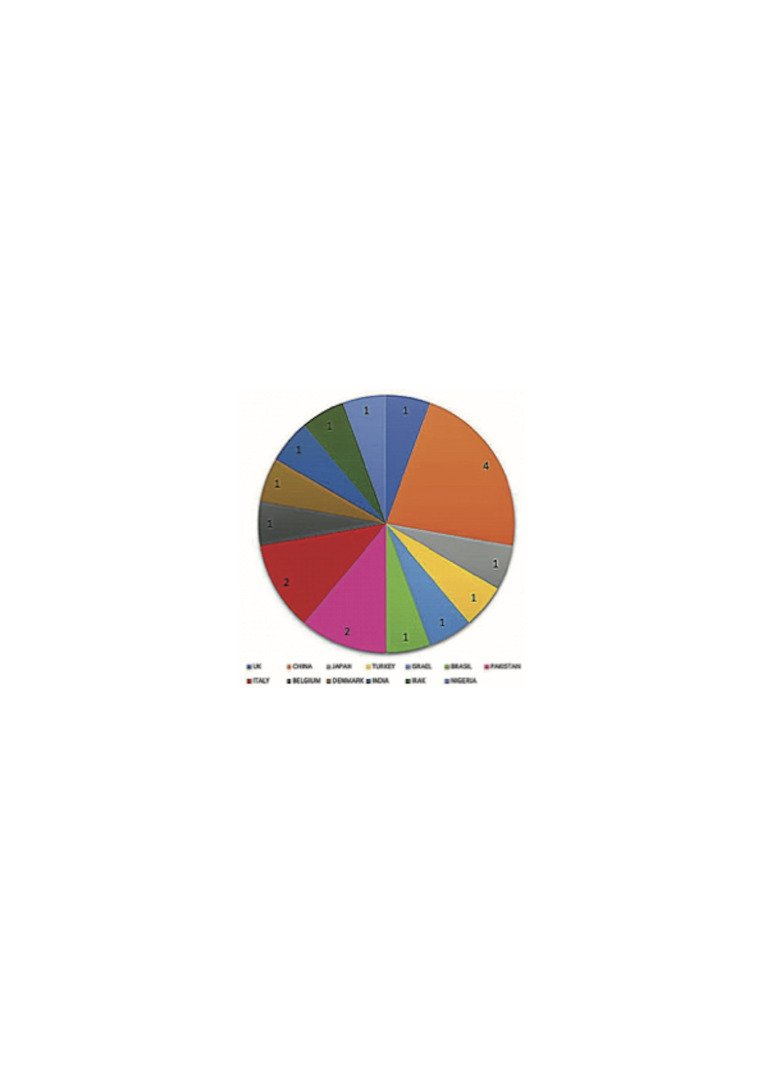
Researchers’ affiliated countries.

Figure 2 and Figure 3 show some of the characteristics of the included studies.

2

3

The largest patient sample was found in the study done by Leung and Cheung (18) in 2016, which included 458 patients who underwent 612 coronectomy procedures. These same authors conducted another study in 2009 (13), which also had a fairly extensive sample: 231 patients with 349 MTMs who were divided into two groups: 178 MTMs were conventionally extracted, and 171 underwent coronectomies.

It is also worth highlighting the many interventions included in Hamad's study (25) in 2024: 422 patients with 438 MTMs, of which 218 were treated by extraction and the remaining 220 by coronectomy.

The results obtained from the different studies and included in this systematic review are summarized in Table 3.

Table 3

Of the 2280 coronectomies performed, 144 (6.22%) were unsuccessful, meaning the complete extraction of the wisdom tooth had to be performed. This was, in the vast majority of cases, a consequence of intraoperative root mobilization.

The most common complication for both procedures was postoperative pain, with an incidence of 15.69% (132/841) in the extraction group and 14.21% (324/2280) in the coronectomy group. Mean pain durations were 3.40 days and 2.61 days, respectively. The visual pain scale (VAS) determined mean pain intensity scores of 1.18 and 1.31 for the extraction and coronectomy procedures.

Regarding IAN injury, 55 cases were obtained in the extraction group, representing 6.53%, of which 5.35% (45/841) were temporary, and 1.18% (10/841) were permanent. However, in the coronectomy group, the incidence of injury was much lower, with 13 (0.56%) of the total number of injuries, of which 0.43% (10/2280) were temporary and 0.13% (3/2280) permanent.

On the other hand, LN injury was the least common complication and was transient in both groups. Transient lingual nerve injury occurred in 0.26% (6/2280) of coronectomy procedures, while only 0.12% (1/841) did so in the extraction group.

The postoperative infection rate was similar in both groups, affecting 3.94% (90/2280) of cases in the coronectomy group and 2.61% (22/841) in the extraction group.

The incidence of dry sockets was quite unequally distributed between the two groups. It was more prevalent in the extraction group, with an incidence of 6.06% (51/841) versus 1.09% (25/2280) in the coronectomy group.

Root migration was found to be the most common complication after coronectomy procedures, occurring mainly during the first 6 months, followed by the interval between 6 and 12 months after surgery, mostly in young patients. The mean root migration was 2.69mm during the first 6 months and 3.2mm at 12 months.

Due to this complication, 3.55% of the coronectomies (81/2280) had to be reintervened after some time, mainly because of this coronal root migration. However, in a small percentage of cases, the reintervention was due to persistent pain and infection.

Of the 16 included studies, only 9 provided pre- or postoperative antibiotic therapy, and 8 prescribed chlorhexidine rinses.

## Discussion

This systematic review examined the complications associated with coronectomy and conventional MTM extraction procedures. It aimed to evaluate and compare both techniques and determine the clinical effectiveness and reliability of coronectomy as a surgical alternative for MTM while minimizing the risk of IAN injury.

To analyze the different articles studied, the discussion was organized into the following sections:

Failed coronectomy rate

The results support the idea that coronectomy of lower wisdom teeth close to the IAN is a safe technique. Our systematic review reveals a mean failure rate of 6.22%, with the study by Renton et al. (12) followed by Kang et al. (17) presenting the highest failure rate, 38.3% and 16.36%, respectively. Renton et al. (12) concluded that both female gender and molars with conical roots significantly increased the risk of intraoperative root mobilization, requiring root extraction, and considered the coronectomy treatment failed. On the other hand, Kang et al. (17) found that in their failed coronectomies the most repeated pattern was vertically or distoangularly impacted wisdom teeth. Likewise, Simons et al. (27), in their clinical trial, determined that, when dealing with both vertical and distoangular inclinations with risk of IAN injury, there was no clear preference on the part of the surgeons regarding the treatment to be performed approximately half chose coronectomy procedures. In contrast, the other half declined for extraction procedures. However, in mesioangular wisdom teeth or with Pell &amp; Gregory III B impactions, coronectomy was the procedure chosen by the majority of surgeons.

Similar results to our study are also presented in the systematic review by Dalle Carbonare et al. (28) in 2017, with an average incidence of failure in coronectomies of 7%.

Risk of nerve injury

Given the high risk of injury to the IAN that exists when extracting MTMs in positions close to or direct contact with the mandibular canal, alternative techniques such as coronectomy have emerged, whose main objective is to minimize the likelihood of nerve damage.

Our review includes 16 studies, 8 comparing the coronectomy technique with conventional total extraction.

Regarding neurological impairment of the IAN, our results show that the incidence of nerve injury after coronectomy is much lower than after conventional extraction, 0.56% versus 6.53%, consistent with results previously reported in the literature. In their systematic review, Long et al. (1) evaluated four studies of coronectomy versus total extraction and indicated that the total extraction group was almost 10 times (1/0.11) more likely than the coronectomy group to suffer an IAN injury. Similar results were also obtained by Peixoto et al. (29) in their meta-analysis, with a prevalence of IAN injury of 7.93% in cases of complete extraction and an only incidence of 0.99% in coronectomies.

Five of the studies in our systematic review (12 , 13 , 17 , 25 - 26) concluded that successful coronectomy of the MTM is significantly safer (p&lt;0.05) than conventional total extraction when the MTM roots show radiographic signs of proximity to the mandibular canal. Renton et al. (12), Kang et al. (17) and James et al. (26) did not have any cases of paresthesia in their group of patients who underwent successful coronectomies, whereas they did experience IAN injury in the conventionally treated patients (18.6%; 10.9% and 14.7% respectively). Moreover, permanent IAN involvement was also much lower in the coronectomy group, accounting for 0.13% compared to 1.18% in the conventional extraction group. All these data confirm again, as Moreno-Vicente et al. (3) concluded in their bibliographic review, that coronectomy is a safe procedure and an adequate technique for preventing IAN injury.

On the other hand, the incidence of LN injury was very low in both groups. Only two of the 16 studies included, those by Pedersen et al. (22) and Mukherjee et al. (11), reported 6 cases of LN paresthesia in patients treated by coronectomy, which represents a total involvement of 0.26%. Of the patients who underwent conventional extraction, only Yan et al. (24) had 1 case of injury (0.12%), and it was also transient.

However, it should be noted that other factors, such as the surgeon's experience, patient age, preexisting diseases, the surgical technique used, the depth of impaction of the MTM, or the radiographic relationship of the MTM roots to the mandibular canal, influence the success of the procedure. This also leads us to the need for good presurgical planning, constantly assessing the probability of injury to the adjacent anatomical structures with a pre-procedure panoramic radiograph. Furthermore, it would be advisable to request a CBCT or TC whenever there are radiographic warning signs associated with nerve damage, such as the intimate relationship of the roots with the IAN canal, deviation, narrowing, and interruption of the canal's white line, or obscuration of the roots themselves, since only with a CBCT or TC is it possible to determine the proper relationship of the MTM roots with the mandibular canal (5 , 30 - 34). However, as far as the course of the LN is concerned, it is not predictable and can only be detected preoperatively by magnetic resonance imaging, which is not reasonable considering the low incidence of LN damage (35).

Postoperative complications

The main postoperative complications that may be found after coronectomy procedures, and generally in the short term, are pain, infection, alveolitis, and root migration.

Postoperative pain was the most common complication suffered in both groups, the coronectomy group (14.21%) and the extraction group (15.69%), with a very similar mean incidence in both groups in our review. The results obtained in the different studies analyzed are pretty heterogeneous. Renton et al. (12) and Leung and Cheung (13) recorded in their randomized clinical trials a significantly (p&lt;0.05) higher postoperative pain involvement in patients undergoing conventional extraction of MTM; moreover, Kang et al. (17) in their study concluded that the duration of pain was also longer in the extraction group. However, the case-control study by Hatano et al. (14) showed significantly greater pain involvement in the coronectomy group. The studies by Yan et al. (24) and James et al. (26) evaluated postoperative pain intensity using the VAS scale and presented similar results in both groups, although they were slightly higher in the coronectomy group. On the other hand, neither Hamad (25) in his prospective study, nor Cervera-Espert et al. (34) in their systematic review and meta-analysis, found statistically significant differences between coronectomy and conventional extraction regarding postoperative pain.

In our analysis, postoperative infection had a low incidence overall in both groups, although it was slightly higher in the coronectomy group (3.94%) compared to the extraction group (2.61%). Much higher results were reported in the study by Nowak et al. (35), with an incidence of infection of 9% for coronectomy.

Comparing the results obtained in both procedures, it is observed that infection affected the complete extraction group in a greater extent in the studies of Leung and Cheung (13) and Hatano et al. (14), 6.7% and 3.39%, respectively. In comparison, infection fell mainly in the coronectomy group in the studies of Renton et al. (12) (5.2%), Cilasun et al. (15) (1.14%), and Yan et al. (24) (10.9%).

Analyzing in more detail the incidence of infection in the group treated by coronectomy, it is worth noting that the authors who did not provide pre- or postoperative antibiotic therapy to their patients (12 , 13 , 18 , 22 , 24) had the highest infection rates, ranging from a minimum of 2.9% to a maximum of 11.7%. Authors who provided postoperative antibiotics (11 , 15 - 17 , 19 , 20 , 23 , 25) counted lower incidences as outcomes, ranging from 0% (11 , 17 , 20 , 23) to 2.85% (19) in all studies analyzed, except Hamad's study (25), whose incidence was 6.4%. Kang et al. (17), who provided their patients with postoperative antibiotics for 3 days, obtained no cases of infection in either of the 2 study groups. In contrast, Yan et al. (24), who did not provide antibiotics or analgesics to their patients, obtained the highest postoperative infection incidence values in both groups, the coronectomy and the extraction one.

A dry socket is among the most frequent complications arising after the extraction of an impacted wisdom tooth, with a frequency of 1-4% in regular extractions and 20-30% in cases of retained MTM (36). Seven articles included in our systematic review compare the incidence of dry sockets in conventional extraction (6.06%) versus coronectomy (1.09%). The results clearly show that coronectomy reduces the incidence of dry sockets. The rates of dry socket cases in the extraction group are significantly higher than those of coronectomy in the studies by Leung and Cheung (13) (2.8% vs. 0%), Hatano et al. (14) (8.47% vs. 1.96%) and Hamad (25) (10.5% vs 4.5%), and are higher, although not significantly, in the studies by Cilasun et al. (15), Kang et al. (17) and Yan et al. (24). Only in isolation, the survey by Renton et al. (12) did present a higher incidence of alveolitis in the coronectomy group compared to the extraction group, 12.1%, and 9.6%, respectively, without statistical significance. Torres-Lagares et al. (37) demonstrated through a clinical trial the high level of efficacy of chlorhexidine gel (placed intraalveolarly before suturing) or mouthwash (in concentrations between 0.12% and 0.2%) when used pre- and postoperatively for the prevention of alveolitis, reducing its incidence by up to 50%.

Generally, in coronectomy procedures, the rate of dry socket observed is not usually higher than the incidence of dry socket in cases of complete removal of MTM. However, by definition, there really cannot be cases of dry sockets in teeth treated by coronectomy since, as the roots are not entirely removed, there is no empty socket as such (9).

Root migration and reintervention

Root migration or delayed MTM root eruption was the most frequent post-surgical complication after coronectomy procedures. Eleven of the studies (11 - 14 , 16 , 17 , 19 - 22 , 25) in our review reported the incidence of root migration, obtaining figures ranging from 2.33% (13) to 100% (19) of cases; at the same time, five of them (11 , 13 , 16 , 17 , 25) also evaluated the distance (in millimeters) migrated by the roots at different periods, generally at 3, 6, 12 and 24 months post-extraction. Leung and Cheung (38), in a prospective study analyzing the long-term pattern of root migration, determined that the migration rate was highest during the first 6 months after coronectomy (91.1%) and that it decreased thereafter, reaching a plateau 2 years after the procedure and remaining similar until 5 years postoperatively (with less than 5% migration between 3 and 5 years post-coronectomy). Most roots migrated in a coronal direction. Age was an influential factor in root migration; younger patients were associated with significantly more root migration during the first six postoperative months than older patients. They found no relationship between the presence of root migration and gender, eruption stage, or type of MTM impaction at any of the periods evaluated.

All studies included in the review came to the same conclusion: Root migration occurs mainly in the first 6 months and is more prevalent in young patients. However, some authors, such as Kang et al. (17), Peixoto et al. (29), and Goto et al. (39), determined that female gender was a predominant factor in migration. Furthermore, Kang et al. (17) concluded that root morphology was the most relevant factor in root migration, and they also reported a statistically significant inverse correlation between root eruption and vertical and distoangular impactions.

Reintervention is only indicated when symptoms or root exposure appear (40). Most studies' leading causes of reintervention were root exposure in the oral cavity due to root migration, infection, pain, and incomplete crown removal, causing enamel retention. Fifteen of the studies in our systematic review detailed the rates of MTM requiring reintervention (11 - 25), ranging from 0% (12) to 9.37% (21), with a mean of 3.55% total involvement in our study. The results of our review show a low incidence of reintervention, even lower than the ones of other reviews, such as that of Barcellos et al. (40), with a retreatment rate of 5.1%. Furthermore, since most MTM roots migrate coronally, separating from the IAN canal, the risk of nerve damage due to reoperation is extremely low (41).

Finally, it is also worth mentioning the technique of germectomy or early extraction of third molars, proposed by other authors as a procedure to prevent nerve injuries and postoperative complications. It consists of extracting the germ of the third molar when it is still developing, and only the crown and a third or fewer roots are formed (42). The procedure is usually performed between 14 and 17 years old, being at this stage when the risk of injury to the IAN and LN is most unlikely (due to the relationship between the germ of the wisdom tooth and these nerves is practically nonexistent), compared to the risk entailed by later conventional extraction (43). In 1995, Chiapasco et al. (44) conducted a clinical trial comparing germectomy with late extraction of MTM. They grouped the 868 patients of the study into three groups based on their age: Group A (9-16 years), group B (17-24 years) and group C (over 24 years). Finally, they determined that age influenced the incidence of postoperative complications, with these being significantly lower in groups A and B, with results of 2.6% and 2.8%, respectively, and with no neurological complications in either group. In group C, complications affected 7.4% of the MTMs, and several cases of IAN and LN injuries were recorded, although with one exception, all were transient. However, despite being considered a promising technique, it remains controversial. This makes it necessary to carefully analyze the cost-benefit of each case and follow the indications for which it was initially proposed (morphostructural alterations, orthodontic space gain by distalizing molars, severe bone-dental discrepancy, tooth bud dysplasia or pathological processes in the mandible) until future studies provide further evidence supporting its efficacy. Although this technique was not fully explored in our systematic review, we believe it is essential always to consider all available options when planning a successful MTM removal treatment.

Limitations of the study

In summary, although this systematic review provides valuable information on the safety and efficacy of coronectomy as an alternative technique to complete extraction in MTM surgery, it is essential to acknowledge the limitations of the included studies.

First, the heterogeneity of study designs and the outcome measures used in the included studies, such as pain or root migration, differed, which may have influenced the results.

Second, some of the studies had small sample sizes and short follow-up periods, so the statistical power of the results may not be sufficient to confirm our conclusions.

Further clinical studies with longer follow-up periods are needed to better understand the long-term risks associated with coronectomy, such as root migration or the need for reintervention.

## Conclusions

The reviewed studies conclude that coronectomy is an effective alternative technique for managing MTM with a risk of IAN injury, as this procedure tends to have a lower complication rate than complete wisdom tooth extraction surgery.

Patients treated with coronectomy had a significantly lower incidence of IAN lesions than those treated with extraction. LN lesions were low in both groups, with no statistically significant differences. Coronectomy also reduced the incidence of postoperative pain and dry socket. The infection rate was similar in both groups.

Root migration was the most common complication associated with coronectomy, occurring most frequently during the first six months and in younger patients. However, in most cases, it had little clinical impact, and the reoperation rate was very low.

The rate of failed coronectomies was low, and the risk of nerve injury in these cases was similar to the one of conventional extraction procedures.

This demonstrates that coronectomy is a low-risk technique and a viable alternative for MTMs with roots very close to the IAN, although further clinical trials with larger sample sizes and more long-term data are still needed.

## Figures and Tables

**Table 1:** Table Risk of Bias Assessed by the Joanna Briggs Institute Critical Appraisal Tools for Prevalence Studies.

	Q1. Was the sample frame appropriate to address the target population?	Q2. Were study participants sampled in an appropriate way?	Q3. Was the sample size adequate?	Q4. Were the study subjects and the setting described in detail?	Q5. Was the data analysis conducted with sufficient coverage of the identified sample?	Q6. Were valid methods used for the identification of the condition?	Q7. Was the condition measured in a standard, reliable way for all participants?	Q8. Was there appropriate statistical analysis?	Q9. Was the response rate adequate, and if not, was the low response rate managed appropriately?	% YES/RISK
Renton et al. (2005) [12]	Y	Y	Y	Y	Y	Y	Y	Y	Y	100% / L
Leung and Cheung. (2009) [13]	Y	Y	Y	Y	Y	Y	Y	Y	Y	100% / L
Hatano et al. (2009) [14]	Y	Y	Y	Y	Y	Y	Y	Y	Y	100% / L
Cilasun et al. (2011) [15]	Y	Y	Y	Y	N	Y	Y	N	Y	77.8% / L
Frenkel et al. (2015) [16]	Y	Y	Y	Y	Y	Y	Y	Y	Y	100% / L
Kang et al. (2019) [17]	Y	Y	Y	Y	Y	Y	Y	Y	Y	100% / L
Leung and Cheung. (2016) [18]	Y	Y	Y	Y	Y	Y	Y	Y	Y	100% / L
Mendes et al. (2020) [19]	U	Y	U	Y	Y	Y	Y	Y	Y	77.8% / L
Cosola et al. (2020) [20]	Y	Y	Y	Y	Y	Y	Y	Y	Y	100% / L
Agbaje et al. (2015) [21]	Y	Y	Y	Y	N	Y	Y	N	Y	77.8% / L
Pedersen et al. (2018) [22]	Y	Y	Y	Y	Y	Y	Y	Y	Y	100% / L
Mukherjee et al. (2016) [11]	U	Y	U	Y	N	Y	Y	N	Y	55.5% / M
Monaco et al. (2023) [23]	Y	Y	Y	Y	N	Y	Y	N	Y	77.8% / L
Yan et al. (2020) [24]	Y	Y	Y	Y	Y	Y	Y	Y	Y	100% / L
Hamad (2024) [25]	Y	Y	Y	Y	Y	Y	Y	Y	Y	100% / L
James et al. (2023) [26]	Y	Y	Y	Y	Y	Y	Y	Y	Y	100% / L

Y: Yes, N: No, U: Unclear, L: Low, M: Moderate, H: High.

**Table 2:** Table General characteristics of the included studies.

Articles	Study Design	Location	Diagnostic Radiography	Nº of Patients (C*/E)	Follow-up	

Renton et al. (2005) [12]	RCT	UK	OPG	128 patients(C*:94 teeth / E:102 teeth)	25 months	

Leung and Cheung. (2009) [13]	RCT	Hong Kong	OPG	231 patients(C*:171 teeth / E:178 teeth)	24 months	

Hatano et al. (2009) [14]	Case-control study	Japan	OPG and TC	220 patients(C*:102 teeth / E:118 teeth)	13,5 months	

Cilasun et al. (2011) [15]	Case-control study	Turkey	OPG and TC	120 patients(C*:88 teeth / E:87 teeth)	6-30 months	

Frenkel et al. (2015) [16]	RCS	Israel	OPG and CBCT	173 patients(C*:185 teeth / E:--)	12 months	

Kang et al. (2019) [17]	PCS	China	OPG and CBCT	92 patients(C*:55 teeth / E:55 teeth)	36 months	

Leung and Cheung. (2016) [18]	PCS	Hong Kong	OPG	458 patients(C*:612 teeth / E:--)	60 months	

Mendes et al. (2020) [19]	PCS	Brazil	OPG and CBCT	21 patients(C*:35 teeth / E:--)	12 months	

Cosola et al. (2020) [20]	PCS	South Korea and Italy	OPG and CBCT	130 patients (C*:130 teeth / E:--)	36-84 months	

Agbaje et al. (2015) [21]	PCS	Belgium	OPG and CBCT	64 patients(C*:96 teeth / E:--)	12 months	

Pedersen et al. (2018) [22]	PCS	Denmark	OPG and CBCT	191 patients(C*:231 teeth / E:--)	12-144 months	

Mukherjee et al. (2016) [11]	PCS	India	Periapical intraoral radiography	18 patients(C*:20 teeth / E:--)	24 months	

Monaco et al. (2023) [23]	PCS	Italy	OPG and CBCT	94 patients(C*:116 teeth / E:--)	36-120 months	

Yan et al. (2020) [24]	PCS	China	CBCT	121 patients(C*:91 teeth / E:49 teeth)	6 months	

Hamad (2024) [25]	PCS	Irak	OPG and CBCT	422 patients(C*:220 teeth / E:218 teeth)	24 months	

James et al. (2023) [26]	RCT	Nigeria	Periapical intraoral radiography and OPG	68 patients(C*:34 teeth / E:34 teeth)	12 months	


RCT: Randomized controlled trial. RCS: Retrospective cohort study. PCS: Prospective cohort study. OPG: Orthopantomography. TC: Computed tomography. CBCT: Cone beam computed tomography. E: Extraction. C*: Coronectomy. --: Not included in the study.

**Table 3:** Table Summary of the data obtained from the studies included in the systematic review.

Articles	Pts.(nº)	Extractions(nº teeth)	Coronectomies(nº teeth)	Pharmacological Treatment	Injury of IAN		Injuryof LN		Postoperat.Pain		Incidence of Infection		Incidence of Dry Socket		Failed vs. Successful Coronectomies	Reinterv.	Root Migration
					Ext	Cor*	Ext	Cor*	Ext	Cor*	Ext	Cor*	Ext	Cor*			
Renton et al. (2005) [12]	128	102	94	Preoperative rinse with CHX.	19(18.6)T:17P:2	0	0	0	22(21.5)	8(13.8)	1(0.98)	3(5.2)	10(9.6)	7(12.1)	F:36 (38.3)S:58 (61.7)	0	5/58(8.62)<2mm
Leung and Cheung. (2009) [13]	231	178	171	After surgery: Paracetamol and codeine for 3 days.	9(5.1)T:6P:3	1(0.65)T:1	0	0	102(57.3)	65(41.9)	12(6.7)	9(5.8)	5(2.8)	0	F:16 (9.4)S:155 (90.6)	1(0.65)	3 months: 1.9mm (62.2)12 months: 2.9mm (11.5)24months: 3.1mm (2)
Hatano et al. (2009) [14]	220	118	102	-	6(5.08)T:3P:3	1(0.98)T:1	0	0	8(6.78)	19(18.6)	4(3.39)	1(0.98)	10(8.47)	2(1.96)	F:5 (4.9)S:97 (95)	5(4.9)	87/102(85.29)
Cilasun et al. (2011) [15]	120	87	88	Postoperative antibiotics and CHX rinses.	2(2.29)T:2	0	0	0	0	1(1.14)	0	1(1.14)	1(1.15)	0	F:2 (2.23)S:86 (97.7)	1(1.14)	-
Frenkel et al. (2015) [16]	173	-	185	Postoperative antibiotics for 1 week.	-	1(0.54)T:1	-	-	-	16(8.65)	-	2(1.08)	-	-	F:10 (5.4)S:175 (94.6)	6(3.24)	6 months: 2.2mm (41)12 months: 12.9mm (3.2)
Kang et al. (2019) [17]	92	55	55	Postoperative antibiotics for 3 days.	6(10.9)T:4P:2	0	-	-	Days:3.40	Days:2.61	0	0	2(5.45)	1(1.82)	F:9 (16.36)S:46 (83.63)	5(9.09)	50/55 (90.9)3 months: 2.19mm6 months: 2.91mm12 months: 3.15mm36 months: 3.19mm
Leung and Cheung. (2016) [18]	458	-	612	Postoperative analgesics (paracetamol and codeine).	-	1(0.16)T:1	-	0	-	191(31.2)	-	18(2.9)	-	1(0.16)	F:20 (3.3)S:592 (96.7)	20(3.3)	-
Mendes et al. (2020) [19]	21	-	35	1g amoxicillin 1h before surgery; 8mg dexamethasone 2h before and 12h after surgery. CHX 0.12% rinses before and after surgery and 500mg paracetamol every 6h for 3 days.	-	1(2.85)T:1	-	-	-	17(48.6)	-	1(2.85)	-	-	F:3 (8.6)S:32 (91.4)	2(5.71)	35(100%)
Cosola et al. (2020) [20]	130	-	130	Preoperative rinses with CHX 0.2% and amoxicillin 1g. Postoperative amoxicillin for 5 days.	-	0	-	0	-	0	-	0	-	0	F:15 (11.53)S:115 (88.46)	10(7.69)	31(23.85)
Agbaje et al. (2015) [21]	64	-	96	-	-	0	-	0	-	4(4.16)	-	4(4.16)	-	4(4.16)	F:9 (9.37)S:87 (90.62)	9(9.37)	14(14.58)
Pedersen et al. (2018) [22]	191	-	231	Paracetamol 1g + ibuprofen 400mg and CHX for 5 postoperative days.	-	5(2.2)T:3P:2	-	5(2.2)T:5	-	0	-	27(11.7)	-	-	F:8 (3.5)S:223 (96.5)	8(3.5)	191(97.45)
Mukherjee et al. (2016) [11]	18	-	20	Amoxicillin 500mg, Metronidazole 400mg and Paracetamol 500mg. All 3 times a day for 3 postoperative days.	-	0	-	1(5.0)T:1	-	3(15.0)	-	0	-	0	F:2 (10.0)S:18 (90.0)	0	5(25.0)1-2mm in 24months
Monaco et al. (2023) [23]	94	-	116	Preoperative: Rinse with CHX and antibiotics 1h before.Postoperative: Ibuprofen, CHX and antibiotics for 4 days.	-	0	-	0	-	-	-	0	-	-	-	6(5.17)	Between the 6-12 months postsurgery. After 24 months < 5% of cases.
Yan et al. (2020) [24]	121	49	91	No medication was given in either group.	0	2(2.19)T:2	1(2.04)T:1	0	VAS:1.84	VAS:1.99	5(10.2)	10(10.9)	NS	NS	F:2 (2.19)S:89 (97.80)	1(1.09)	-
Hamad (2024) [25]	422	218	220	Postoperative: CHX for 7 days and Antibiotic and Paracetamol for 3 days.	8(3.7)T:8	1(0.5)P:1	-	-	VAS:NS	VAS:NS	-	14(6.4)	23(10.5)	10(4.5)	F:7 (3.2)S:213 (96.8)	7(3.2)	163 (74)3 months: 2.37mm6 months: 3.35mm12months: 3.85mm24months: 3.89mm
James et al. (2023) [26]	68	34	34	Postoperative: Ibuprofen for 3 days; Amoxicillin for 5 days and CHX rinses for 10 days.	5(14.7)T:5	0	-	-	VAS:0.53	VAS:0.63	-	-	-	-	-	-	-

(): Data in %, Pts.: Patients, Postoperat.: Postoperative, Reinterv.: Reintervention, Ext: Extraction, Cor*: Coronectomy, T: Transitory, P: Permanent, F: Failed, S: Successful, VAS: Visual Pain Scale, NS: There is no significant difference between both groups, CHX: Clorhexidine.

## Data Availability

The datasets used and/or analyzed during the current study are available from the corresponding author.
